# Application of Smart Wearable Fitness Equipment and Smart Health Management Based on the Improved Algorithm

**DOI:** 10.1155/2022/1654460

**Published:** 2022-08-21

**Authors:** Haibo Cao

**Affiliations:** Xinyang Normal University, Xinyang, Henan 464000, China

## Abstract

IoT technology has scientific, advanced, and practical characteristics in wearable fitness equipment and artificial intelligence health management. On this basis, this paper discusses the intelligent health management system based on IoT technology. By building an IoT health management system, the design principles and algorithm design of wearable fitness equipment are introduced around the device, and various noises that affect the accuracy of the equipment and how to better filter out various noises are also analyzed. The authors selected cloud service platforms and also investigated and analyzed the application prospects of wearable fitness equipment in the community. From the perspective of real-time monitoring, scientific management, and intelligent decision-making, we ensure high-quality, standardized, scientific, efficient, and accurate wearable fitness equipment applications and intelligent health management. In addition to simply viewing IoT technology as an information dissemination platform, IoT technology can also expand many other functions. According to the survey results of this article, 90% of people will use the APP with the function of sports and fitness in social media. One of the most concerned issues in the current society is the application of social media in national fitness activities.

## 1. Introduction

The 21st century is the 5.0 era of the information society, because human society has begun to rely mainly on computers, using modern intelligent technology as the main means to work and live. Smart wearable fitness equipment is mainly used in road runners, so this article investigates and analyzes the applications of road runners and smart wearable fitness equipment [1, 2]. The survey results show that among road runners, the main considerations for purchasing and using smart wearable devices are product type, quality, functional effects, price, brand, etc. At present, there is not much investment in smart wearable devices and insufficient publicity in the market [3]. People do not know enough about the devices, and there are not many road runners buying smart wearable devices. The purchase of smart wearable devices is also related to the age and work status of road runners. Specific influencing factors include the consumption concept of road runners, the use experience of road runners, and the quality, function, appearance, and price of smart wearable devices [4]. Although the current sales of smart wearable devices are not impressive, with the development of the times and technological progress, road runners' acceptance of smart wearable devices will become higher, and the demand for devices will naturally change accordingly [5].

Since the current level of technological development cannot better support the application of smart wearable devices, the current application of devices also has some practical application problems [6]. If there are problems, companies can increase the promotion of equipment. For example, they can broadcast advertisements on TV, put up billboards in subway stations, and set up promotion points on running roads; relevant departments should also establish a complete system. The supervision system of smart wearable devices promotes the functional construction of smart wearable devices on the market and the improvement of supporting facilities [7]; the most important thing is to increase scientific research and accelerate the development of science and technology [8]. This article analyzes the application and research status of the Internet in sports and fitness at home and abroad and sorts out and analyzes the existing “Internet plus” related literature, and conducts research on the application of “Internet plus” in sports and fitness [9]. The development of this research conclusion will play a positive role.

## 2. Related Work

This article has consulted a large number of related literature. Literature mainly studies the status quo of physical fitness at home and abroad and the situation of network fitness [10]. According to the research results, it compares the fitness situation at home and abroad and also compares network fitness and offline fitness. The development status of, puts forward the advantages and disadvantages of each fitness method, and then proposes each specific solution. Literature mainly investigates whether the Internet of Things has a good promotion effect on the sports fitness industry, and how to apply the Internet of Things to sports fitness and the principles and methods that must be followed when using it [11]. Literature mainly studies smart wearable devices, researches the market prospects and technological prospects of the devices, and points out that one of the most important functions of smart wearable devices is to detect the user's movement and the body's physical function, and believes wearing smart wearable devices can help users exercise scientifically and reasonably [12]. Literature shows that the era of big data is inseparable from the Internet [13]. To truly enter the era of big data, the Internet must be integrated into all aspects of people's lives and work. Literature introduced a new fitness model integrated with the Internet by analyzing fitness clubs, and explained how the new fitness model will develop in the future and how to better integrate the two [14]. Literature discusses how to promote the deeper integration of “Internet+” into the fitness industry [15]. To integrate the two more deeply, first, the people's awareness of using smart wearable devices should be strengthened. Only when buyers are willing to purchase the devices can the industry continue to develop; second, it is necessary to further promote the concept of fitness and improve the fitness of all people. Awareness; then, strengthen the further application and development of “Internet plus” in the fitness industry; finally, the government should introduce some policies in a timely manner to encourage innovation, encourage the development and sales of smart wearable devices, and encourage the masses to actively exercise and exercise. Chinese Academy of Sciences has begun to study sensor networks and its related technologies. In fact, not only China is studying sensor networks, but some other countries are also studying sensor networks, and the research on sensor networks is almost simultaneous. Up to now, research studies on sensor networks have begun to take shape, such as the development and progress of miniature sensors, wireless smart sensors, and mobile base stations.

One of the important components of the sports industry is the fitness industry. The concept of a healthy lifestyle has been deeply rooted in the hearts of the people, so the masses' acceptance and recognition of sports wearable electronic devices is also increasing. In the beginning, smart wearable devices were just words, but after the continuous development of history, smart wearable devices can finally become tangible entities, develop into today's small computers, and have a different impact on people's daily lives.

## 3. Application of Wearable Sports Fitness Equipment Based on the Internet of Things

### 3.1. Design Principles of Wearable Sports Fitness Equipment

The motion state of the human body is generally indicated by the attitude angle. After understanding the attitude angle, the next step is the attitude coordinate system. The attitude coordinate system corresponds to the attitude angle. In order to no longer express the attitude angle and the attitude coordinate system abstractly, we take a car as an example to introduce the attitude angle and attitude coordinate system to make it more concrete. The attitude angle includes roll angle, pitch angle, and heading angle yaw. The heading angle yaw is the attitude angle rotating around the *Z* axis; the pitch angle is the attitude angle rotating around the *Y* axis; the roll angle roll is the attitude angle rotating around the *X* axis.

First, we introduce the geographic coordinate system to explain the posture coordinate system. The definition of geographic coordinate system is: Choose a point *O* on the ground as the origin; the *X*-axis direction is in the horizontal plane and points to any direction; the *Z*-axis is perpendicular to the ground and points to the center of the Earth; the *Y*-axis is in the horizontal plane and perpendicular to the *x*-axis, and the *Y*-axis direction is ruled by the right hand determine.

The basic rotation matrix obtained by basic rotation of each axis is as follows:

(1) $$\begin{array}{c} C_{2}^{b}=\left(\begin{array}{ccc} \cos\gamma & 0 & -\sin\gamma \\ 0 & 1 & 0\\ \sin\gamma & 0 & \cos\gamma \end{array}\right),\\ C_{n}^{1}=\left(\begin{array}{ccc} \cos\;\psi & -\sin\;\psi & 0\\ \sin\;\psi & \cos\;\psi & 0\\ 0 & 0 & 1 \end{array}\right),\\ C_{1}^{2}=\left(\begin{array}{ccc} 1 & 0 & 0\\ 0 & \cos\;\theta & \sin\;\theta \\ 0 & -\sin\;\theta & \cos\;\theta \end{array}\right). \end{array}$$

The conversion method to convert the geographic coordinate system to the attitude coordinate system is as follows:

(2) $$\begin{array}{c} C_{n}^{b}=C_{n}^{1}C_{1}^{2}C_{2}^{b}\\ =\left(\begin{array}{ccc} \cos\;\psi & -\sin\;\psi & 0\\ \sin\;\psi & \cos\;\psi & 0\\ 0 & 0 & 1 \end{array}\right)\left(\begin{array}{ccc} 1 & 0 & 0\\ 0 & \cos\;\theta & \sin\;\theta \\ 0 & -\sin\;\theta & \cos\;\theta \end{array}\right)\left(\begin{array}{ccc} \cos\;\gamma & 0 & -\sin\;\gamma \\ 0 & 1 & 0\\ \sin\;\gamma & 0 & \cos\;\gamma \end{array}\right). \end{array}$$

Then, the conversion relationship between the two is as follows:

(3) $$\begin{array}{c} \left(\begin{array}{c} x_{n}\\ y_{n}\\ z_{n} \end{array}\right)=C_{b}^{n}\left(\begin{array}{c} x_{b}\\ y_{b}\\ z_{b} \end{array}\right), \end{array}$$

where *C*_*n*_^*b*^ is the direction cosine matrix that transforms the geographic coordinate system and the attitude coordinate system. Below, we will use quaternions. Quaternions refer to four elements. The formula is as follows:

(4) $$\begin{array}{c} Q\left(q_{0},q_{1},q_{2},q_{3}\right)=q_{0}+q_{1}i+q_{2}j+q_{3}k. \end{array}$$

The quaternion also has a certain relationship with the attitude matrix, the relationship is as follows:

(5) $$\begin{array}{c} C_{b}^{n}=\left[\begin{array}{ccc} q_{0}^{2}+q_{1}^{2}-q_{2}^{2}-q_{3}^{2} & 2\left(q_{1}q_{2}-q_{0}q_{3}\right) & 2\left(q_{1}q_{3}+q_{0}q_{2}\right)\\ 2\left(q_{1}q_{2}+q_{0}q_{3}\right) & q_{0}^{2}-q_{1}^{2}+q_{2}^{2}-q_{3}^{2} & 2\left(q_{2}q_{3}-q_{0}q_{1}\right)\\ 2\left(q_{1}q_{3}-q_{0}q_{2}\right) & 2\left(q_{2}q_{3}+q_{0}q_{1}\right) & q_{0}^{2}-q_{1}^{2}-q_{2}^{2}+q_{3}^{2} \end{array}\right]. \end{array}$$

Correspondingly, the quaternion also has a certain relationship with the attitude angle as shown below:

(6) $$\begin{array}{c} \text{roll}=\arctan\left[2\left(q_{2}q_{3}+q_{0}q_{1}\right),1-2\left(q_{1}^{2}+q_{2}^{2}\right)\right],\\ \text{pitch}=\arcsin\left[2\left(q_{0}q_{2}-q_{1}q_{3}\right)\right],\\ \text{yaw}=\text{arctan}\left[2\left(q_{1}q_{2}+q_{0}q_{3}\right),2\left(q_{0}{}^{2}+q_{2}{}^{2}\right)-1\right]. \end{array}$$

In normal life, we will take the number of steps as the amount of exercise and use the heart rate range to illustrate the exercise intensity. At the same time, the value of the heart rate will also be used to judge anaerobic exercise and aerobic exercise. For how to judge, we will use the target heart rate to judge. In sports medicine, the target heart rate is the range of the heart rate of the human body during aerobic exercise, and this range of heart rate is certain. When the heart rate during exercise is not within this range, it is considered that the exercise we are doing is anaerobic exercise. In sports medicine, the target heart rate is fixed, but in real life, the target heart rate is not fixed. The target heart rate varies from person to person. The calculation formula for the target heart rate is as follows:

(7) $$\begin{array}{c} \text{THR}=\left(220-\text{Age}-\text{Static heart}\right)*\left(60\text{\%}-80\text{\%}\right)+\text{Static heart.} \end{array}$$

### 3.2. Community Fitness Gait Data Preprocessing and Algorithm Design

The inertial sensor is used this time, which is characterized by light weight, small size, easy to carry, and low cost. However, when the sensor collects information, it will be interfered by itself and the outside world, such as external burst noise and its own high frequency noise; these interferences will definitely affect the algorithm design, so that the collected gait data are not accurate. In fact, not only the sensor will be interfered but also trapped at the current level of technology, no matter what kind of sensor will be interfered by itself and external noise. Therefore, if you want to further develop smart wearable devices, you must remove noise. The noise removal method will be introduced, analyzed, and compared in the following paragraphs; then, the most suitable method for noise removal will be selected.

**Figure 1:**
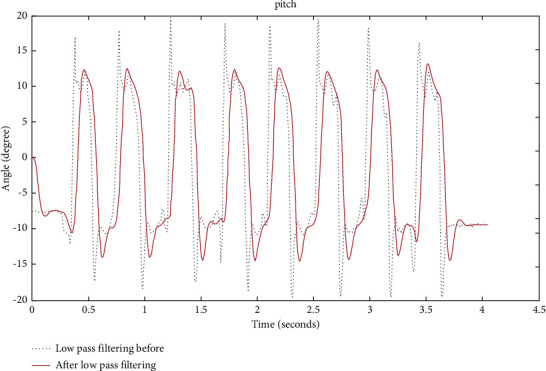
Comparison before and after low pass filtering.

When extracting the gait information of the human body when walking or running, as mentioned in the previous paragraph, there will be high-frequency noise generated by the sensor itself. This high-frequency noise includes uniform noise and Gaussian noise. Generally, IIR filters and FIR filters are used in engineering to remove this type of noise. The former filter has a simple structure and fewer operations, which can better filter high-frequency noise. The passband and transition band of the filter will affect the filtering effect of the filter. If you want to strengthen the filtering effect, you need to find a way to make the passband of the filter as flat as possible and speed up the attenuation of the transition band. The order can affect the passband and transition band of the filter, and increasing the order can make the filtering effect of the filter better. However, at the same time, the increase in the order makes the time delay of the filter increase, so you must be careful when choosing the order of the filter, and you need to consider the timeliness. The comparison before and after low pass filtering is as shown in Figure 1.

**Figure 2:**
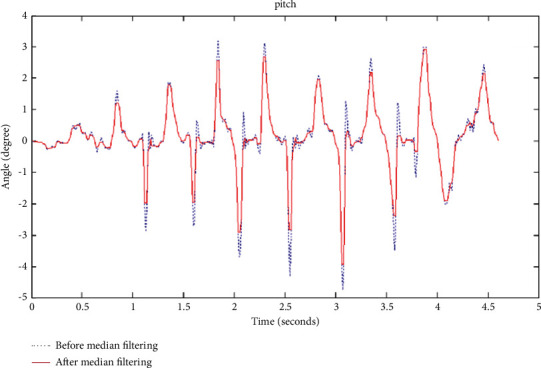
Comparison chart before and after median filtering.

Comparing the two line graphs in Figure 1, it is found that the original data are smoother after low-pass filtering. After the noise of the equipment itself can be filtered out, it is time to find a way to filter out the noise. For how to remove the external noise, one method is to use the median filter method, which is a nonlinear smoothing filter method. The filtering is convenient, and the algorithm is simple, and the filtering range can be set by yourself. The core of the median filtering method is the value of the window size. If the pulse width of the external noise is within the range of the window value, then the external noise at this time can be filtered out. Once the window value is less than the pulse width of the external noise, the noise beyond the window value cannot be filtered out. The window value is equivalent to a limited range, the noise within the range can be removed, and the part that exceeds the limited range cannot be filtered out. Of course, the selection of the window value is also very important. If the window value is too small, the noise that can be filtered is very limited. However, once the window value is too large, it may not only filter out noise but also filter out valid data. Comparison of the chart before and after median filtering is as shown in Figure 2.

**Figure 3:**
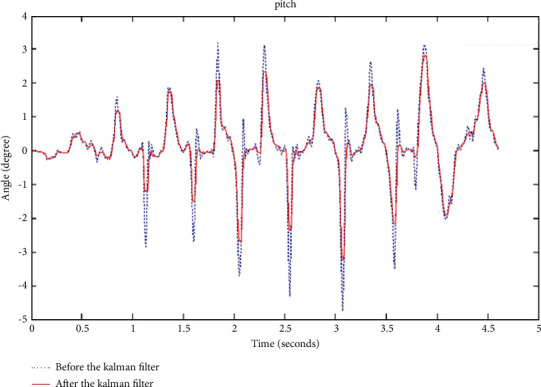
Comparison of before and after the Kalman filter.

It can be seen from Figure 2 that after median filtering, the filtered data are smoother than the original data. It has advantages and disadvantages. Median filtering can only filter out the noise contained in slow-changing variables. This is because the median filter needs to determine the sampling values of multiple points before and after. Therefore, if the noise in the variable that changes quickly is filtered out, the delay phenomenon of the median filter will be more obvious. Assuming that the Kalman filter time is long enough, the initial value will not affect the estimated value of the filter. Comparison of before and after the Kalman filter is as shown in Figure 3.

As can be seen from Figure 3, the effect of Kalman filtering is very good, but because the Kalman filtering algorithm is an iterative algorithm, this algorithm requires a high level of data processing by the processor, so the calculation of Kalman filtering is large.

The Kalman filter algorithm has five main equations:

A priori estimate:

(8) $$\begin{array}{c} X\left(k|k-1\right)=AX\left(k-1|k-1\right)+BU\left(k\right). \end{array}$$

The most important element in the Kalman filter algorithm is the angle, but the angle cannot be measured directly. To get the angle value, you must use a gyroscope. The gyroscope has static drift. The static drift refers to the output value of the gyroscope when it is stationary. The angular velocity measured by the gyroscope at a certain moment is subtracted by the static drift value of the gyroscope and then multiplied by the time at that moment. The sum of the angles at a moment is the angle value at this moment. However, the angle value obtained by this calculation method is not accurate. This is because there will be errors in the measurement of the gyroscope, so the angle value we calculated is only an approximate value. Therefore, the angle prediction formula at the current moment is as follows:

(9) $$\begin{array}{c} \text{Angle}={\text{Angle}}^{\prime }+\left(\text{Gyro}-Q_{\text{bias}}\right)*dt. \end{array}$$

In fact, the static drift value of the gyroscope changes. However, we believe that the drift value is constant when making predictions, namely:

(10) $$\begin{array}{c} Q_{\text{bias}}=Q_{{\text{bias}}^{\prime }}. \end{array}$$

Write the two equations in the following matrix form:

(11) $$\begin{array}{c} \left[\begin{array}{c} \text{Angle}\\ Q_{\text{bias}} \end{array}\right]=\left[\begin{array}{cc} 1 & -\text{d}t\\ 0 & 1 \end{array}\right]\left[\begin{array}{c} {\text{Angle}}^{\prime }\\ Q_{{\text{bias}}^{\prime }} \end{array}\right]+\left[\begin{array}{c} \text{d}t\\ 0 \end{array}\right]\text{Gyro.} \end{array}$$

Prediction of covariance matrix:

(12) $$\begin{array}{c} P\left(k|k-1\right)=\text{AP}\left(k-1|k-1\right)A^{\prime }+Q. \end{array}$$

Because the angle does not affect the drift noise, the covariance between the two is zero, that is:

(13) $$\begin{array}{c} \left\{\begin{array}{c} \mathrm{cov}\left(\text{Angle},Q_{\text{bias}}\right)=0,\\ \mathrm{cov}\left(Q_{\text{bias}},\text{Angle}\right)=0. \end{array}\right. \end{array}$$

We substitute and simplify to the following formula:

(14) $$\begin{array}{c} p\left(k|k-1\right)=\left[\begin{array}{cc} \left(a-c\right)*\left(dt-b\right)*dt+D\left(\text{Angle}\right), & \left(b-d\right)*dt,\\ \left(c-d\right)*dt, & d. \end{array}\right]. \end{array}$$

Calculation of the Kalman gain is as follows:

(15) $$\begin{array}{c} Kg\left(k\right)=P\left(k|k-1\right)\frac{H^{\prime }}{\left(HP\left(k|k-1\right)H^{\prime }+R\right)}. \end{array}$$

Correction through the Kalman gain:

(16) $$\begin{array}{c} X\left(k|k\right)=X\left(k|k-1\right)+Kg\left(k\right)\left(Z\left(k\right)-H\left(k|k-1\right)\right). \end{array}$$

We update the covariance matrix:

(17) $$\begin{array}{c} P\left(k|k\right)=\left(I-Kg\left(k\right)H\right)P\left(k|k-1\right). \end{array}$$

Smart wearable devices will not only be interfered by their own noise and external noise but sometimes also have occasional pulse interference. It is more effective to eliminate occasional interference by limiting the filter. Because the interference occurs by accident, this shows that the sampled value is relatively regular under normal circumstances, so once accidental interference occurs, the sampled value will deviate from this law. Therefore, the limiting filter method will set an allowable maximum deviation value, and set the maximum deviation value A by summarizing the difference between two adjacent sample values in the past. When the device collects a new sample value, it is set to new_value, Then, the value detected last time is last_value, if new_value-last_value < A; then, the value detected this time is judged to be valid. Otherwise, the value detected this time is judged to be invalid, and the value detected last time is used to replace the detected value this time.

**Figure 4:**
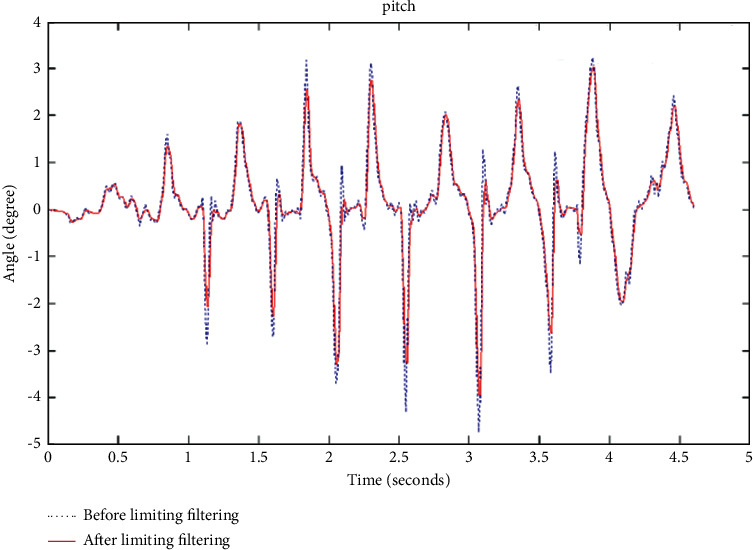
Comparison before and after clipping filtering.

The limiting filtering algorithm is also relatively simple, which is suitable for filtering the noise contained in the variable that changes quickly, to make up for the deficiency that the median filter can only filter the noise contained in the variable that changes slowly. The comparison before and after clipping filtering is as shown in Figure 4.

The algorithmic characteristics of the limiting filter make it not have too much influence on the original data, and only filter out the accidental noise interference, which can also be seen from the figure above.

Limiting filter, Kalman filter, and median filter can filter impulse noise well. Limiting filtering compares the difference between two adjacent points with the empirical value. If the difference is greater than the empirical value, the data of the previous point will be used to replace the data of the current point. The algorithm of limiting filtering is simple and it is very timely to filter out noise. However, it is only suitable for filtering out sudden noise interference; the filtering effect of the Kalman filter is the best among the three methods, but there are too many steps, the algorithm is more complicated, and the iterative calculation in the Kalman filter algorithm affects the data of the processor. The processing level is very demanding; the median filter algorithm is simple, and the filter range can be set by itself. The core of the median filter method is the value of the window size. If the pulse width of the external noise is within the range of the window value, then the external noise at this time can be filtered out. Once the window value is less than the pulse width of the external noise, the noise beyond the window value cannot be filtered out. After median filtering, the filtered data are smoother than the original data. It has advantages and disadvantages. Median filtering can only filter out the noise contained in slow-changing variables. This is because median filtering requires multiple points before and after a certain point. The value can determine the filtering value at this point. Therefore, if the noise in the variable that changes quickly is filtered out, the delay phenomenon of the median filtering will be more obvious. Considering that this article is to design a device that can detect the number of steps in real-time without delay, and the current Chinese processors are not enough to support the complex iterative calculations in the Kalman filter algorithm, this article chooses the limiting filter to filter in addition to impulse noise.

In the design of the gait algorithm, because it completes the function of step counting, aerobic exercise, and anaerobic exercise are not distinguished, but the extracted gait data are analyzed. The step-counting function here is also inseparable from the attitude angle. We can extract the posture based on the energy of the posture angle to complete the step counting function, or set the threshold to extract the posture to complete the step counting function. This article sets two thresholds *T*1 and *T*2, and based on this, two step counting points *a* and *b* are set. Point a represents the intersection of threshold *T*1 and attitude angle and point *b* represents the intersection of threshold *T*2 and attitude angle.

(18) $$\begin{array}{c} \left\{\begin{array}{cc} \left[\text{pitch}\left(t_{a}-1\right)-T1\right]*\left[\text{pitch}\left(t_{a}+1\right)-T1\right] & ≺0,\\ \text{pitch}\left(t_{a}+1\right)-T1 & ≻0, \end{array}\right.\\ \left\{\begin{array}{cc} \left[\text{pitch}\left(t_{b}-1\right)-T2\right]*\left[\text{pitch}\left(t_{b}+1\right)-T2\right] & ≺0,\\ \text{pitch}\left(t_{b}+1\right)-T2 & ≻0. \end{array}\right. \end{array}$$

The main biological factors that can affect the level of energy expenditure are gender, age, and weight. Due to the different physiological characteristics of individuals, even if everyone does the same intensity of exercise, the energy consumed by people is different. This article uses the method of combining indirect calorimetry and motion sensors, combined with the user's gender, age, weight and other biological factors, and uses smart wearable devices to collect the steps of road runners, and compare the number of steps with road runners. The weight and gender of the person are substituted into the improved oxygen uptake model, because the energy consumption value is equal to the product of the oxygen uptake and the breathing entropy oxygen heat price, so the oxygen uptake model can be used instead of the energy consumption model. There are two calculation models below to calculate oxygen uptake, one is a linear model and the other is an exponential model.

Weight is the weight, the unit is kg; when the user's gender is female, *G*1 = 0, *G*0 = 1; when the gender is male, *G*1 = 1, *G*0 = 0; speed is the speed, the unit is km/h; *Y* is the camera. The amount of oxygen is in ml/min.

The linear model is as follows:

(19) $$\begin{array}{c} Y=\left(0.66+0.117\right)*\left(\text{speed}-\text{0.}002\text{weight}\right). \end{array}$$

The index model is as follows:

(20) $$\begin{array}{c} Y=0.034*\left(\text{weight}-0.00018\right)*\left({\text{weight}}^{2}+0.503\right)*e^{\left(0.178*\text{speed}\right)}-\left(1.57G_{1}-1.66G_{0}\right). \end{array}$$

**Figure 5:**
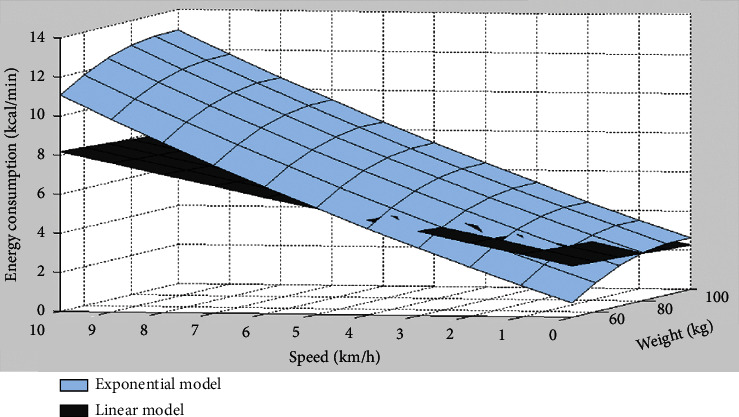
Model of speed, weight, and energy consumption during aerobic exercise.

It can be seen from the two model formulas that gender has no effect on the linear model; for the exponential model, gender is a constant difference, so this article only selects the male exponential model and the linear model for comparison. Taking weight and speed as independent variables and energy consumption as dependent variables, the model diagram can be obtained by simulation with MATLAB. The model of speed, weight, and energy consumption during aerobic exercise is as shown in Figure 5.

### 3.3. Technology Selection of the IoT Embedded System

A necessary condition for smart wearable devices is to connect to the Internet. Connecting to the Internet means that a cloud platform is required to serve the device. An important basis for measuring whether the cloud service platform is easy to access, expand, and maintain is the cloud service system. The measurement of cloud service system is whether the cloud service system has strong system expansion capabilities. After investigation, it is found that Alibaba Cloud ECS server can design its own IoT development platform for designers to use, so this article uses Alibaba Cloud ECS server for design work. Users can log in remotely through virtual machines to connect to and manage Alibaba Cloud servers. The Alibaba Cloud Server has two operating systems, Linux and Windows. The security and stability of the Linux system is relatively strong, and the deployment of data services can be completed professionally. The Windows system has comprehensive functions, but it is commonly used on computers, so this article chooses the Aliyun server of the Linux system. Alibaba Cloud single server configuration is as shown in Table 1.

**Figure 6:**
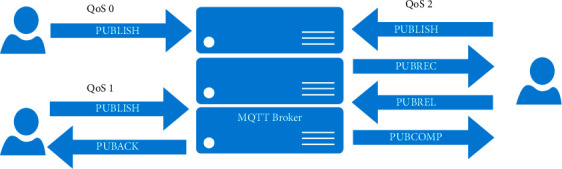
MQTT Broker and user data interaction method.

Table 1 shows the configuration of a single server in Alibaba Cloud. We use the above table to complete the deployment of the application in this article. There are currently three commonly used IoT communication protocols: XMPP, CoAP, and MQTT. It is necessary to select a protocol that meets the characteristics of this article. When in a resource-constrained environment, CoAP performs better in application, but it is more likely to produce a packet loss rate. MQTT Broker and user data interaction method is as shown in Figure 6.

The necessary condition for a stable connection between the client and the Broker is that MQTT and KeepAlive maintain a long connection. If the client cannot receive the information from the server within a certain period of time, the client will disconnect from the server at this time, indicating that there is a connection problem at this time. The transmission method of MQTT has relatively high requirements for system space. It is precisely because of the high requirements for system space that MQTT can guarantee the quality of transmission and efficient service. While ensuring the quality of service, it does not take the cost of saving space, so the Internet of Things MQTT is more suitable for the application layer protocol of the development framework. Because Alibaba Cloud is based on a virtual server, there is no need to build a computer room or purchase a physical server. Therefore, it is more efficient to use MQTT for application deployment. Comparison of three IoT communication protocols is as shown in Table 2.

### 3.4. Prospects of Wearable Sports Fitness Equipment in the Community

The current smart wearable fitness products can not only record various data, to a certain extent, guide people to perform scientific exercises but they can also record the human body's food intake, calorie consumption, exercise volume and sleep status that day. In terms of product types, future smart wearable devices will tend to diversify, linking smart wearable products with daily necessities, such as incorporating devices into jewelry or other daily necessities. The role of intelligent wearable devices used by road runners is as shown in Table 3.

Road runners are the main consumers of smart wearable products. According to the survey, road runners are more concerned about the fashion and beauty of smart wearable devices. Therefore, the design of smart wearable devices in the future must be unique and stylish. For example, the equipment can be made into jewelry products such as earrings and necklaces or it can be embedded in clothes and shoes, which can be used as ornaments or incorporated into daily necessities. The abovementioned improvements are just the icing on the cake for smart wearable devices. In the final analysis, we should return more energy and attention to the quality and functions of the device. After all, road runners buy this type of equipment because of the multiple functions of the device. Product. Therefore, the future development direction of smart wearable technology must be to pursue the authenticity and accuracy of measurement data, and the diversity of functions, while at the same time, it can also take into account beauty and fashion. The impact of wearable devices on road runners is as shown in Table 4.

At present, we can start from two aspects to make the measured data more accurate: one is to increase the sensor, but the increase of the sensor will increase the volume of the device, and also reduce the endurance of the device; the other is to develop more advanced algorithms. The progress of the sensor will make the measurement data of the sensor more accurate. Defects in wearable device applications are as shown in Table 5.

Smart wearable devices can only be used normally when connected to the Internet. Connecting to the Internet means that there will be hackers who will send phishing URLs. Some unprepared users are likely to click on the URL to open suspicious links or pictures. At this time, hackers also achieved the goal.

## 4. Artificial Intelligence Health Management Based on the Internet of Things

### 4.1. Construction of an Artificial Intelligence Health Management System

The purpose of this paper is to realize a set of equipment health management system based on the Internet of Things. To achieve this goal involves two key technologies: Internet of Things technology and fault prediction and health management technology. If fault prediction and health management technologies are used, the following three key issues will be faced:

First, how to build a knowledge base for fault prediction and health management, and on this basis, study the health monitoring algorithms of each device to make the detection and monitoring results more accurate.

Second, how to use the advantages of the Internet of Things to collect real-time data to ensure long-term uninterrupted communication between nodes and nodes and hosts in the Internet of Things.

Third, whether the system platform can adapt to the diversification of equipment. The system platform needs to be well adapted to the various changes of the equipment, and have a certain degree of scalability, such as integrating fault detection algorithms and fault pre-diagnosis algorithms into the system in the form of plug-ins, and achieve the communication between plug-ins and the communication between the plug-in and the program, etc.

**Figure 7:**
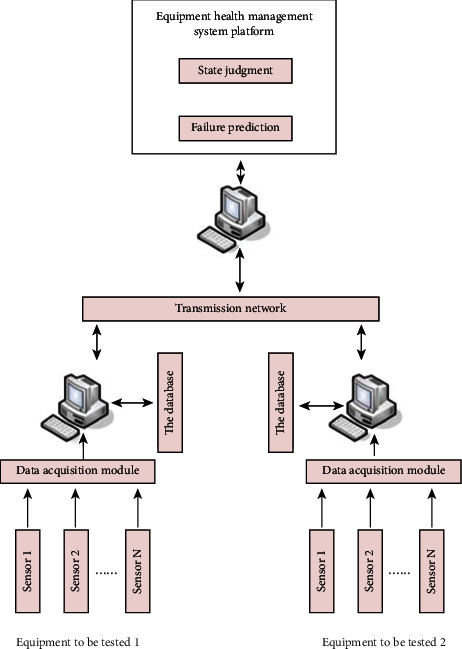
Overall scheme of the system.

The system scheme will be described and analyzed in detail below. The system solution model is shown in Figure 7.

### 4.2. The Application Status of the Internet of Things and 5G in Community Artificial Intelligence Health Management

The community is another relatively extensive living space for people in addition to the family, and now, China is vigorously promoting the nationwide fitness program, and various communities are also beginning to build and improve physical fitness infrastructure. Therefore, the development of China's sports and fitness industry needs to make full use of the community and combine with the advantages of the Internet of Things fitness to provide more community residents with a more scientific fitness method. The scope and target of the community is larger than that of the family, which also shows that the community is more oriented towards the general public. At present, some communities in China have begun to gradually establish fitness-instructing websites. These websites are for residents of various communities and are used to guide residents in proper fitness. On these websites, residents can inquire about sports information anytime, anywhere and can also find related fitness programs and provide solutions to emergencies. Of course, there are two flowers, one for each table. For the dissemination of fitness information not only online but also offline, in people's practical lives, we will also build fitness activity bases to spread fitness knowledge for community residents offline.

General communities have special activity centers, but it is not realistic to purchase large-scale fitness equipment in economically underdeveloped areas. However, in order to facilitate residents to perform physical fitness activities and perform fitness activities in a scientific environment, the economy is underdeveloped. Multimedia activity rooms have been established in local communities. Multimedia fitness guidance is used to disseminate sports fitness videos through “Internet+” technology. In the multimedia activity rooms, sports fitness videos will be played through projection technology. Community residents can be more scientific after watching fitness videos effectively.

### 4.3. Development Strategy of Wearable Sports Fitness Equipment

Smart wearable devices must ensure that the product is reasonably priced and of high quality. Based on the various data of the detected road runners, through big data analysis, it will push each targeted knowledge to different users, so as to provide scientific and reasonable guidance, such as designing a reasonable running route, environmental prompts, nutrition matching, and running plan for road runners. Because the main body of using smart wearable devices is road runners, the functions of smart wearable devices meet the needs of road runners.

So how to build a complete supporting facilities for smart wearable devices, we can start from the following two aspects. Starting from the equipment itself, we should make smart wearable devices better serve the road runners. The functions of the equipment can be extended, and the accuracy of the equipment can be deepened, so that the road runners must need smart wearable devices. Starting from the crowd, we can raise the crowd's fitness awareness, make people aware of the concept and necessity of scientific fitness, so as to stimulate people's desire to buy smart wearable devices. Of course, the improvement of smart wearable devices can not only start from the above two aspects, but the above two aspects must be relatively easy to start, and it is relatively easy to make improvements.

## 5. Conclusion

The main body of this paper is a smart wearable device. Around the device, it introduces the design principles and algorithm design of wearable fitness equipment. It also analyzes various noises that affect the accuracy of the equipment and how to better filter out various noises, and then selected the cloud service platform of the device, and also investigated and analyzed the prospects of wearable fitness equipment in the community, as well as the problems that may be encountered in the future, and solutions and solutions. But overall, the future of smart wearable devices will be a blue ocean. First, from the social perspective, enterprises should focus on learning from advanced management experience, actively innovate, and be brave to innovate; second, from the perspective of government departments, government departments should establish and improve a relatively systematic and scientific supervision system to ensure that the industry environment is clean and free of gray areas. Moreover, government departments must vigorously cultivate relevant talents and integrate various sports resources to promote the active and healthy development of the industry.

## Figures and Tables

**Table 1:** Alibaba Cloud single server configuration.

Specification family	Processor model	nCPU	Main frequency of processor (GHz)	RAM (GB)	Intranet send and receive packets	Intranet bandwidth (GHz)
General network enhanced sn2ne	Intel Xeon	2vCPU	2.5	2	30万PPS	2.5
	E5-2682v4					

**Table 2:** Comparison of three IoT communication protocols.

	XMPP	CoAP	MQTT
Based on agreement	TCP	UDP	TCP
Message method	Request response	Request response	Publish and subscribe
Data structure	XML	Binary	Binary
Suitable for the environment	High network quality	Resource constraints	Universal network
Internet broadband requirements	Larger	Extremely small	Extremely small
Equipment support	Difficulty	Easy	Easy

**Table 3:** The role of intelligent wearable devices used by road runners.

Effect	Number of samples	Selected ratio (%)	Sort
Detect body indicators	587	59.59	1
Motion tracking	557	56.55	2
Collect data and share running results	508	51.57	3
Make a plan to improve fitness	495	50.25	4
Monitoring and reminder service	345	35.03	5
Environmental recognition	253	25.69	6
Entertainment and fashion oriented	240	24.37	7
Not so useful	67	6.80	8

**Table 4:** The impact of wearable devices on road runners.

Factor	Number of samples	Selected ratio (%)	Sort
Effect	730	74.11	1
Product quality	670	68.02	2
Price	620	62.94	3
Portability	608	61.73	4
Brand	497	50.46	5
Other	163	16.55	6

**Table 5:** Defects in wearable device applications.

Problem	Number of samples	Selected ratio (%)	Sort
Accuracy of recorded data	785	79.70	1
Privacy security issues	558	56.60	2
Material problem	470	47.72	3
Insufficient practicality	415	42.13	4
Other	207	21.02	5

## Data Availability

The data used to support the ﬁndings of this study are available from the corresponding author upon request.
